# New Agilent platform DNA microarrays for transcriptome analysis of *Plasmodium falciparum* and *Plasmodium berghei* for the malaria research community

**DOI:** 10.1186/1475-2875-11-187

**Published:** 2012-06-08

**Authors:** Björn F C Kafsack, Heather J Painter, Manuel Llinás

**Affiliations:** 1 Lewis-Sigler Institute for Integrative Genomics, Princeton University, Princeton, NJ, 08544, USA

## Abstract

****Background**:**

DNA microarrays have been a valuable tool in malaria research for over a decade but remain in limited use in part due their relatively high cost, poor availability, and technical difficulty. With the aim of alleviating some of these factors next-generation DNA microarrays for genome-wide transcriptome analysis for both *Plasmodium falciparum* and *Plasmodium berghei* using the Agilent 8x15K platform were designed.

****Methods**:**

Probe design was adapted from previously published methods and based on the most current transcript predictions available at the time for *P. falciparum* or *P. berghei*. Array performance and transcriptome analysis was determined using dye-coupled, aminoallyl-labelled cDNA and streamlined methods for hybridization, washing, and array analysis were developed.

****Results**:**

The new array design marks a notable improvement in the number of transcripts covered and average number of probes per transcript. Array performance was excellent across a wide range of transcript abundance, with low inter-array and inter-probe variability for relative abundance measurements and it recapitulated previously observed transcriptional patterns. Additionally, improvements in sensitivity permitted a 20-fold reduction in necessary starting RNA amounts, further reducing experimental costs and widening the range of application.

****Conclusions**:**

DNA microarrays utilizing the Agilent 8x15K platform for genome-wide transcript analysis in *P. falciparum* and *P. berghei* mark an improvement in coverage and sensitivity, increased availability to the research community, and simplification of the experimental methods.

## **Background**

Malaria remains one of the most deadly infections to humans worldwide. It is caused by several species in the genus *Plasmodium*, with *Plasmodium falciparum* being the most virulent. The malaria parasite’s complex lifecycle is initiated by an infectious bite from a female anopheline mosquito, injecting sporozoites into the bloodstream and leading to the invasion of liver cells. The subsequent growth of asexual parasites within red blood cells (RBCs) is responsible for pathology. A small percentage of infected RBCs transforms into male and female gametocytes, which can be transmitted to the mosquito vector. After fusion of gametes within the midgut, development proceeds at various sites in the mosquito ending with the invasion of the salivary glands. Upon the vector’s next bloodmeal the life cycle is completed with the injection of salivary gland sporozoites.

While malaria is among the longest studied afflictions of humans, progress towards new therapeutics and vaccines has generally been slow. The past decade has seen significant advances in the fundamental understanding of the parasite’s biology, which in turn has opened new and promising avenues for novel anti-malarial development. This resurgence has been brought about in large part by technological advances that have enhanced the ability to genetically manipulate the parasite [1,2] as well as through the insights provided by the whole genome sequencing of many species and strains of *Plasmodium*[3-6]. A major benefit of genome sequencing has been the ability to assay gene transcription on a genome-wide basis through the use of DNA microarrays, which have allowed probing questions to be asked regarding the transcriptional status at distinct life stages, differences in gene expression between strains, in response to changes in environmental conditions or drug perturbations. Microarrays can also be used to find genetic alterations such as copy number variations or single nucleotide polymorphisms. Despite these possible uses, the DNA microarray has not been broadly utilized for several reasons including the large amount of biological material required, technological impracticality for many research settings, and relatively high cost.

The first DNA microarrays for *P. falciparum* were generated using either sheared genomic DNA [7] or cDNAs [8] and were used to compare differences across asexual blood stages. In the absence of the genome sequence, follow up of potential gene expression differences was pursued on a candidate basis by sequencing of the material spotted on the array. With the completion of the *P. falciparum* strain 3D7 reference genome [3], oligonucleotide-based arrays quickly followed and provided an unprecedented in-depth view of transcriptional changes during asexual development in red blood cells [9,10]. Subsequent studies determined that other strains (HB3, Dd2) exhibited similar transcriptional programs *in vitro*[11] and ultimately these general observations were extended to transcriptional profiles of patient isolates by either profiling parasite mRNA abundance directly from blood [12,13] or after short-term *ex vivo* culturing [14]. Such transcriptional signatures from non-culture adapted parasites have identified subtle and important differences that are the source of on-going research. Other *in vitro* studies have struggled to identify variations in transcriptional profiles under environmental perturbations [15-17], although larger scale efforts examining growth perturbations under dozens of conditions have yielded associations between genes based on transcriptional covariation [18-21]. More detailed studies have examined transcriptional changes associated with differences in cellular adhesion and antigenic variation [15,22]. Ultimately, one of the real powers of DNA microarray analysis will be to characterize transcriptional responses to targeted genetic alterations [23,24].

Since these early studies, other stages of *P. falciparum* development have also been explored using DNA microarrays including sporozoites [25] and various stages of gametocyte maturation [23,26,27]. Furthermore, several efforts have utilized DNA microarrays for genome-wide transcriptional analysis in the rodent models of malaria, *Plasmodium berghei* and *Plasmodium yoelii*. From these efforts, there are reports characterizing ookinete development in *P. berghei*[28,29] and Mikolajczek *et al* have examined oocyst *vs.* salivary gland sporozoites in *P. yoelii*[30]. The liver stage of human malaria parasites has been difficult to analyse transcriptionally. However, microarray studies of *P. yoelii,* or *P. berghei* liver stage infections have characterized both the parasite [31] and host transcriptional programs during this developmental stage [32].

In light of the many insights already gained from this relatively small number of genome-wide experiments, improving the performance and increasing the availability of *Plasmodium* DNA microarray to the research community remains a worthwhile effort.

## **Methods**

### **Microarray design**

Annotated transcripts where downloaded for *P. falciparum* strain 3D7 (PlasmoDB release 7.1) [33] and *P. berghei* ANKA (Sanger Institute, March 2011). These sequences were supplemented with exogenous genes commonly used in *Plasmodium* molecular biology (*hdhfr, bsd, gfp, fkbp*, etc.) and published *P. falciparum* non-protein coding RNAs not included in PlasmoDB at the time [34-38]. Transcripts were first masked for simple repeats using RepeatMasker [39] and then split in half until all fragments were less than 1040nt in size to obtain approximately 15,000 sub-sequences for which a single probe would be designed to achieve near-maximal coverage given the 8x15K array format. These sequences were analysed using OligoBLAST [40] to design 60mer oligonucleotide probes followed by probe selection using OligoRankPick [40] with the following parameters: WBLAST= 6,7,8,9 / WGC = 6,7,8,9 / WSW:1,2 / WLZ = 1 / length = 60 / GC% = 31.4 (see [40] for details). Probes were then BLASTed against the Crick strand of the target transcripts using NCBI BLAST [41] and probes with multiple hits of E-value < 1e^-15^ were marked as non-unique (Additional files 1 and 2). Probes were then randomly distributed across the array (to reduce spatial bias) using the 8x15K format after submission to Agilent Technologies eArray [42]. For this design, no linker sequence was used and any empty features were filled with random duplicate probes. Slides with printed arrays were ordered directly from Agilent Technologies (Santa Clara, CA, USA).

### **Parasite growth and culturing**

*Plasmodium falciparum* strain 3D7 was cultivated *in vitro* using standard techniques [43] with the following modifications. Parasites were grown at 4–5 % haematocrit at 5 % CO_2_/ 6 % O_2_ in RPMI media supplemented with 0.25 % Albumax I (Gibco, San Diego, CA, USA), 0.1 mM hypoxanthine, 2 g/L sodium bicarbonate, 25 mM HEPES pH7.4, and 50 μg/L gentamycin. For assessing transcripts throughout the intra-erythrocytic development, parasites were double synchronized by L-alanine treatment [44] 12 hours apart during the preceding cycle and harvested every six hours throughout the 48-hour cycle, starting approximately three hours post-invasion.

### **RNA extraction**

Cells were harvested from 50 ml of culture suspension by centrifugation at 1,500 rpm for 5 min. Total RNA was extracted and purified using TriZol reagent (Invitrogen, Grand Island, NY, USA) at a volume of 5 ml reagent to 1 ml of packed parasitized erythrocytes as previously described [9]. Quality and quantity of total RNA extracted was assessed by agarose gel electrophoresis and using a ND-1000 (NanoDrop Technologies, Thermo Scientific, Wilmington, DE, USA) then stored at −80 °C for later use.

### **cDNA generation and dye coupling**

Detailed step-by-step protocols can be found in the latest edition of *Methods in Malaria Research* Chapter 2.6 (in preparation) and on the Llinás laboratory website [45]. Single-strand aminoallyl-containing cDNA synthesis and Amersham CyDye-coupling (GE Healthcare, Piscataway, NJ, USA) was carried out as previously described [46]. To eliminate Cy5 degradation by ozone [47], all steps starting with dye resuspension were carried out in an ozone-free environment.

### **Array hybridization and washing**

Final cDNA concentration and dye-incorporation was assessed on a NanoDrop ND-1000 spectrophotometer. These measurements were utilized to calculate the frequency of incorporation and samples that met the manufacturer’s suggested target of 10–20 fmol of dye/ng cDNA were further used for array hybridization. Equal amounts between 50 and 1000 ng of Cy3 and Cy5-labelled cDNA were hybridized on each array for 16 h in a rotating hybridization oven (10 rpm) at 65 °C. Prior to scanning, arrays were washed in 6X and 0.06X SSPE (both containing 0.005 % N-lauryl-sarcosine (Sigma-Aldrich, St. Louis, MO, USA), followed by an acetonitrile rinse. For the 48-hour time-course, cDNA from each time-point was labelled with Cy5 and hybridized to an equal amount of Cy3-labelled cDNA reference pool generated from equal amounts of ring, trophozoite, and schizont stage mRNA.

### **Scanning, data acquisition and analysis**

Arrays were scanned on an Agilent G2505B Microarray Scanner (Agilent Technologies, Santa Clara, CA, USA) with 5 μm resolution at wavelengths of 532 nm (Cy3) and 633 nm (Cy5) using the extended dynamic range (10–100 %) setting. Normalized intensities were extracted using Agilent Feature Extractor Software Version 9.5 employing the GE2-v5_95_Feb07_no_spikein extraction protocol and uploaded to the Princeton University Microarray Database (PUMA.princeton.edu) for analysis.

## **Results & discussion**

### **Array design**

Three main goals provided the impetus for switching DNA microarray platforms from an in-house spotted 70mer array to the Agilent Technologies 60mer SurePrint platform; (1) to improve array performance in terms of the number of features, sensitivity, and inter-array variability, (2) to have the ability to easily and affordably update microarray design as genome annotation improves, and (3) to make *Plasmodium* transcriptome analysis widely available to the research community at-large.

Switching to the Agilent Technologies platform enables researchers with access to a two-color microarray scanner to perform genome-wide transcriptome analysis for *P. falciparum* or *P. berghei* without the need for collaboration or the considerable cost of synthesizing large number of oligonucleotides for spotting and the even greater cost of microarray spotting equipment. Due to Agilent’s manufacturing process any number of slides, from one to hundreds, can be printed and ordered at identical cost per slide. Time from order to delivery varied from three to six weeks.

Unlike the photolithographic manufacturing of Affymetrix arrays, which require expensive masks to print, or spotted oligo-arrays, which required the one-time bulk purchase of synthesized oligonucleotides for a large number of arrays with each redesign ([40] and [48]), Agilent arrays can be redesigned without any additional cost, allowing researchers to alter or update array designs quickly and affordably as the need arises.

Agilent Technologies offers a variety of slide designs with one or more arrays per slide ranging from 15,000 to 1 million features per array. Given the good performance of the previous spotted 10,000-feature array and to keep cost at a minimum, the 8x15K slide format was chosen, which features eight arrays with 15,000+ features on each slide. This provides the added advantage of being able to hybridize multiple samples on the same slide, minimizing variation of hybridization conditions thereby reducing array-to-array variability. In addition, the chosen format is optimal for a full 48-hour intraerythrocytic development cycle (IDC) to be assayed on a single slide when collecting samples every six hours.

For this array design, the annotated transcripts from *P. falciparum* strain 3D7 (PlasmoDB [33] release 7.1) and *P. berghei* strain ANKA (Welcome Trust Sanger Centre, March 2010 Annotation) were used. These sequences were supplemented with exogenous genes commonly used in *Plasmodium* molecular biology (*hdhfr, bsd, gfp, fkbp*, etc.) (Additional files 1 and 2) and for *P. falciparum* published non-protein coding RNAs not included in the PlasmoDB release [34-38]. These transcripts were masked for simple repeats using RepeatMasker [39] and split depending on sequence length to yield ~15,000 sub-sequences, for each of which a single probe would be designed. Transcripts of fewer than 1040 nt were not split, 1041–2080 nt were split into two sub-sequences, 2081–3120 nt were split into three and so forth, with the goal of just under 15,000 probes and 1+ probes for every ~1 kb of transcript length.

These sub-sequences were run through OligoBLAST [40] to generate all possible non-masked 60mers (maximum length for Agilent arrays) and score their uniqueness. From this set the best quality probe was chosen for each sub-sequence using OligoRankPick [40]. The resulting probes were BLASTed against the Crick strand of the initial target transcripts and any probes with hits of E-value < 1e^-15^ were marked as non-unique to indicate the potential for cross-hybridization. Furthermore, several additional handpicked probes for targets of particular interest to this laboratory were also included (e.g. DNA-binding domains of ApiAP2 proteins). This final set of probes was randomly distributed across the 8x15K array (one copy per array) format with the remaining available features filled by random repeats of existing probes. The 8x15K design includes an Agilent Control Grid [42], which serves various functions including manufacturing controls, alignment during feature extraction, and features for spike-in experiments using the Agilent RNA Spike-In Kit. Additionally, a small number of transcripts were repeated 15+ times each across the array for use in further spatial normalization during feature extraction.

The final designs of the new 8x15K *P. falciparum* and *P. berghei* arrays are summarized in Table 1 and details on the 8x15K arrays can be found in Additional files 1 and 2. Briefly, 14,000+ probes were designed for each array, with an average of 2.5+ probes per transcript (Table 1, Additional file 3). Probe specificity was estimated at greater than 97 % for both arrays and over 97 % of transcripts have one or more unique probes. Less than 1 % of transcripts are not represented on these arrays either because they were too short (< 60 nt) or failed to contain a single stretch of 60 nt of non-repeat sequence. Additionally, roughly 2 % of transcripts did not have any unique probes for one of the following reasons: very short (ncRNAs), recent gene duplication (e.g*.* falcipain-2a/b (PF11_0161/PF11_0164) or elongation factor 1a (PF13_0304/PF13_0305)), or belonging to large gene families (*vars*, *rifins*, *BIRs*, etc.). It should be noted that for most genes belonging to such a gene family this does not indicate an absence of unique probes. For example, the *P. falciparum* array contains unique probes for 58 of the 63 *var* genes (*var* gene fragments or *var* pseudogenes were not included in this number but were included when analysing probes for specificity). In total there are unique probes for 5,752 transcripts including 930 transcripts not previously represented on the spotted array design.

**Table 1:** Overview of array features

	**Hu* et al.* Array**	*** P. falciparum* v7.1 8x15K**	***P. berghei* Mar2011 8x15K**
Agilent Design ID (AMADID)	n.a.	037237	038059
GEO Platform Accession	GPL8088	GPL15130	GPL15131
Annotated Transcripts	5,092	5579	5022
Additional ncRNAs	0	257	0
Exogenous Genes	1	14	13
Total Transcripts	5093	5850	5035
Average Probes per Transcript	1.9	2.5	2.9
Genes with 1+ unique probes	4934 (92.1 %)	5752 (97.7 %)	4936 (97.4 %)
Genes with no unique probes	150 (2.8 %)	98 (1.7 %)	99 (1.9 %)
Genes without any probes	271 (5.1 %)	35 (0.6 %)	34 (0.7 %)
Unique Probes	10159 (97.5 %)	14353 (97.1 %)	14464 (98.0 %)
Non-unique Probes	257 (2.5 %)	434 (2.9 %)	299 (2.0 %)
Hand-picked	0	54	0
Repeated Probes	9 (3x3)	345 (23x15)	425 (25x17)
Random Duplicates to fill array	n.a.	22	20
Agilent QC Grid	n.a.	536	536

Feature comparison of the Hu *et al.* in house printed 70mer array [39] and the *P. falciparum/P. berghei* 8x15K Agilent Technologies SurePrint 60mer arrays.

#### Array performance

Performance analysis was limited to the *P. falciparum* array as large quantities of *P. falciparum* total RNA were easily accessible. The *P. berghei* 8x15K array is not expected to differ in terms of array-wide performance measurements as probes were designed using the same methodology. In fact, collaborators have successfully used these arrays for transcript analysis (Andrew Waters, personal communication) validating their utility using similar methods.

**Figure 1:**
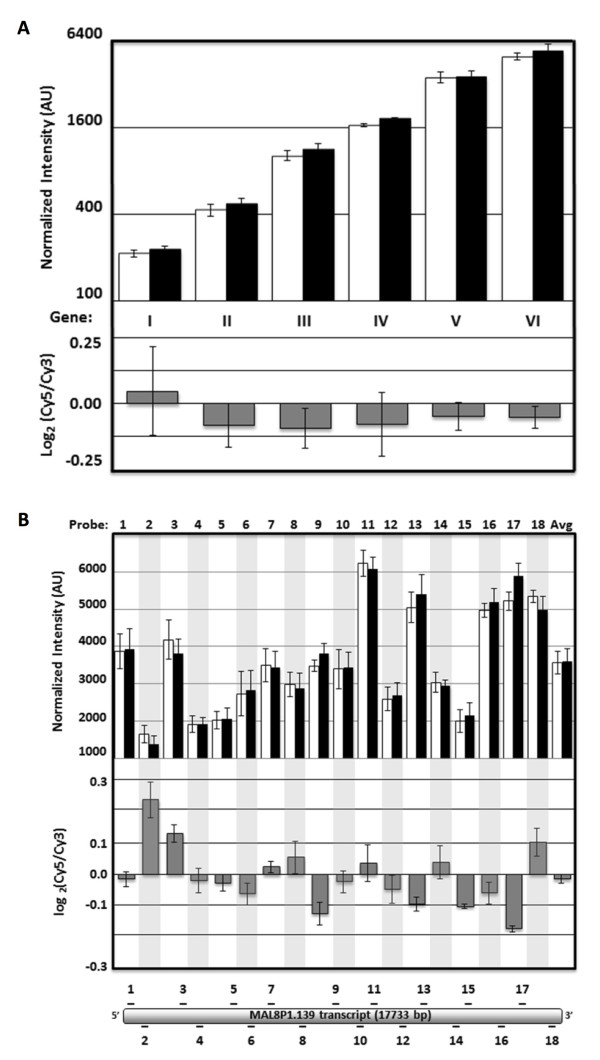
**High overall consistency of log-ratio measurements across a wide range of transcript abundance.** Normalized Cy3 (white), Cy5 (black), and log_2_(Cy5/Cy3) (grey) intensities from triplicate self-hybridization experiments for (**A**) Six genes with average intensities representing a 32-fold range (I = MAL7P1.89, II = PF14_0419, III = PF11_0528, IV = PF07_0118, V = MAL8P1.139, VI = PF11_0506) and (**B**) the 18 probes targeting MAL8P1.139 along with the probe average. (Error bars represent SEM). Non-feature background levels for the 8x15K arrays are very low with 98.6 % of probes yielding both Cy5 and Cy3 signals at least 2.5 standard deviations above background (data not shown). This needs to be taken into account during analysis, as weak non-specific binding of features targeting genes known to not be expressed in some samples occasionally produce very low signal that is nevertheless above non-feature background. In order to assess the minimum amounts of total RNA starting material and dye-labelled cDNA required, cDNA was generated from a pool of total RNA harvested at various stages of the IDC. As little as 500 ng of total RNA starting material yielded 460 ± 13 ng of amino-allyl labelled cDNA and 241 ± 4 ng of dye-coupled cDNA (Additional file 5) with 24 ± 0.4 fmol/ng of dye-incorporation. To test the minimum amount of cDNA required for hybridization, two separate pools of cDNAs with Cy3 and Cy5 were labelled respectively and hybridized a diminishing series of equal amounts from each pool (1,000 ng, 500 ng, 250 ng, 100 ng, 50 ng). Again examining the same set of genes representing a wide abundance range (see Figure 1A), only small differences in the Cy3/Cy5 log ratio measurements was observed across this range of hybridized material with the maximum fold-difference between any two of the 15 measurements for a given gene being 1.35-fold (Figure 2). No significant change in the number of transcripts with signal intensities called as “well above background” by Agilent Feature Extractor Software was observed when hybridizing decreasing amounts material (Additional file 6A). Furthermore, both Cy5 and Cy3 gene signal intensities array-wide correlated very highly (r > 0.96, Additional file 6B) across the dilution series down to 100 ng, and even at 50 ng of hybridized material gene signal intensities matched the 1000ng sample closely (r > 0.82, Additional file 6B). Thus, the amount of dye-coupled cDNA obtained from as little as 500 ng of total RNA is sufficient for performing two to four hybridization experiments.

A cDNA pool derived from an even mixture of ring, trophozoite, and schizont-stage RNA was self-hybridized onto three *P. falciparum* 8x15K arrays to assess inter-probe and inter-array variability. For further in-depth analysis, six genes with coverage by at least 15 probes each were chosen as they represent a wide range of transcript abundance encompassing the full dynamic range of the measurements. Inter-array consistency was good overall for both the mean intensity of each gene (σ ≤ 15 %, Figure 1A top) or each probe individually (σ ≤ 23 %, Figure 1B top). While probe-to-probe intensity can vary significantly across a given gene (Figure 1B top), but has little effect on log-ratio measurements when averaged across probes (Figure 1A bottom) or even when considering individual probes (Figure 1B bottom). When extending this analysis to the entire array, technical reproducibility was good between replicates (Pearson’s r > 0.80) at both the gene and probe level for the log_2_(Cy5/Cy3) ratio as well as for the Cy5 and Cy3 intensities across all probes (Additional file 4). For this reason a common cDNA reference pool should be used when doing multi-sample comparisons of transcript abundance. Furthermore, even for a large transcript such as MAL8P1.139 (17.8 kb), no 3’ bias (Pearson’s r = − 0.36, Figure 1A) was found during cDNA generation.

**Figure 2:**
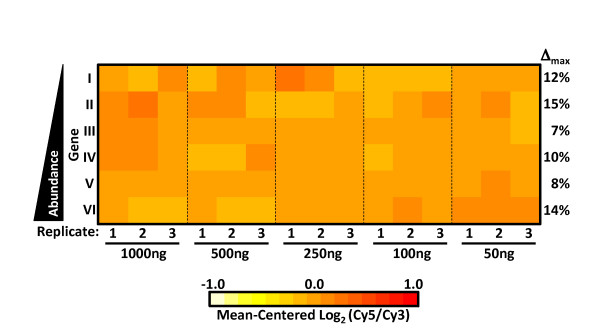
**High consistency of log-ratio measurements across a 20-fold range of hybridized material.** Heat map of mean-centered log_2_(Cy5/Cy3) ratios for six genes representing a 32-fold intensity range (see Figure 1A) across a 20-fold range of hybridized material. For each gene the maximum percent fold-difference (Δmax) between any two of the triplicate averages is also shown. As a final assessment of array performance relative transcript abundances at eight IDC time points were measured at 6-hour intervals. At each time-point *P. falciparum* strain 3D7 total RNA was isolated, reverse transcribed, and Cy5-coupled. These eight samples were each hybridized to one of the eight arrays on a single 8x15K slide, along with an equal amount of Cy3-labelled reference pool. To illustrate correspondence of these results with previously published work, 47 periodically expressed reference genes were chosen that peak in expression within successive three-hour windows [9]. This reference set (available as a table in Additional file 7) can be used for easy visualization of a variety of time-course attributes such as synchrony, progression through the IDC, correct ordering of time-points, etc. As expected, the abundance profiles of these reference transcripts reproduced the characteristic “barber-pole” pattern of the IDC transcriptional cascade (Figure 3) and mirrored existing results at corresponding time points (median Pearson’s = 0.95). [11,49]. The results remained consistent when extending this comparison genome-wide (median Pearson’s r = 0.83).

**Figure 3:**
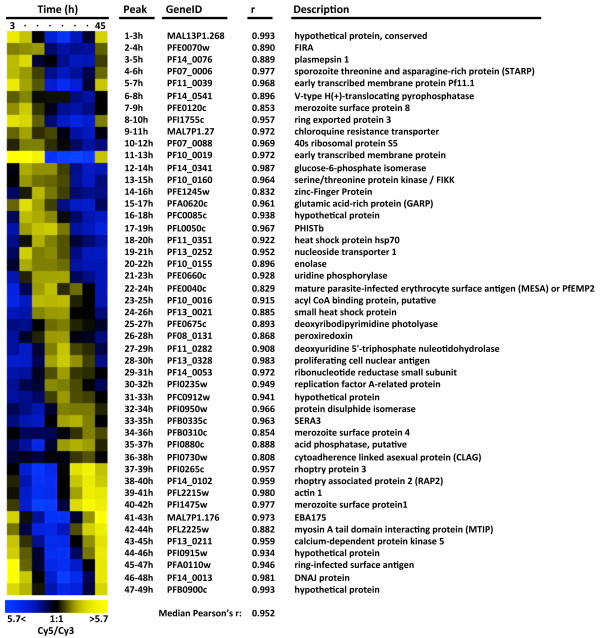
**Periodic gene expression.** Relative mRNA abundance for 47 periodically expressed reference genes at eight time-points covering the intraerythrocytic development cycle of *P. falciparum* strain 3D7. Pearson’s coefficient of correlation (r) is indicated when compared to corresponding time-points of a prior published 3D7 time-course [11].

## **Conclusions**

This paper describes two new long-oligonucleotide arrays for transcriptome analysis in *P. falciparum* and *P. berghei* using the Agilent Technologies SurePrint 60mer 8x15K platform. The advantages of these arrays include 50 % more probes compared to our previous in-house spotted-array, the ability to easily update the array design as genome annotation evolves at no cost, and wide availability to the research community. The new 8x15K *P. falciparum* array was demonstrated to have excellent reproducibility and sensitivity, which allows for transcript analysis with considerably lower amounts of materials compared to previously used methods, thus minimizing the need for high volume samples or RNA/cDNA amplification.

## **Competing interests**

The authors declare they have no conflicts of interest.

## **Authors' contributions**

BK designed the arrays, analysed the data, and contributed equally to the generation of figures and tables. HJP developed the experimental protocols, performed the experiments and contributed equally to the generation of figures and tables. ML contributed to the design of the microarrays and analysis. All authors contributed to experimental design and to writing the manuscript. All authors read and approved the final manuscript.

## Supplementary Material

Additional file 1 Excel file of *P. falciparum* v7.1 8x15K microarray probes. click here for file

Additional file 2 Excel file of *P. berghei* Mar2011 8x15K microarray probes. click here for file

Additional file 3 Figure illustrating coverage for three arrays. Click here for file

Additional file 4 Figures and table describing array-wide technical reproducibility of hybridizations across three replicates. Click here for file

Additional file 5 Table of titration series of total RNA starting amounts, resultant cDNA generation, dye-coupling, and array hybridization. Click here for file

Additional file 6 Table of transcripts well-above background by amount hybridized and table of signal intensity correlation across a self-hybridization dilution series. Click here for file

Additional file 7 List of highly expressed periodic genes used in Figure 3. Click here for file
