# Supplementary material for: New Agilent platform DNA microarrays for transcriptome analysis of Plasmodium falciparum and Plasmodium berghei for the malaria research community
Source: Malar J. 2012 Jun 8;11:187. doi: 10.1186/1475-2875-11-187 (PMC3411454; doi:10.1186/1475-2875-11-187)
Supplement: Additional file 3 — Figure illustrating coverage for three arrays. [file 1475-2875-11-187-S3.pdf]

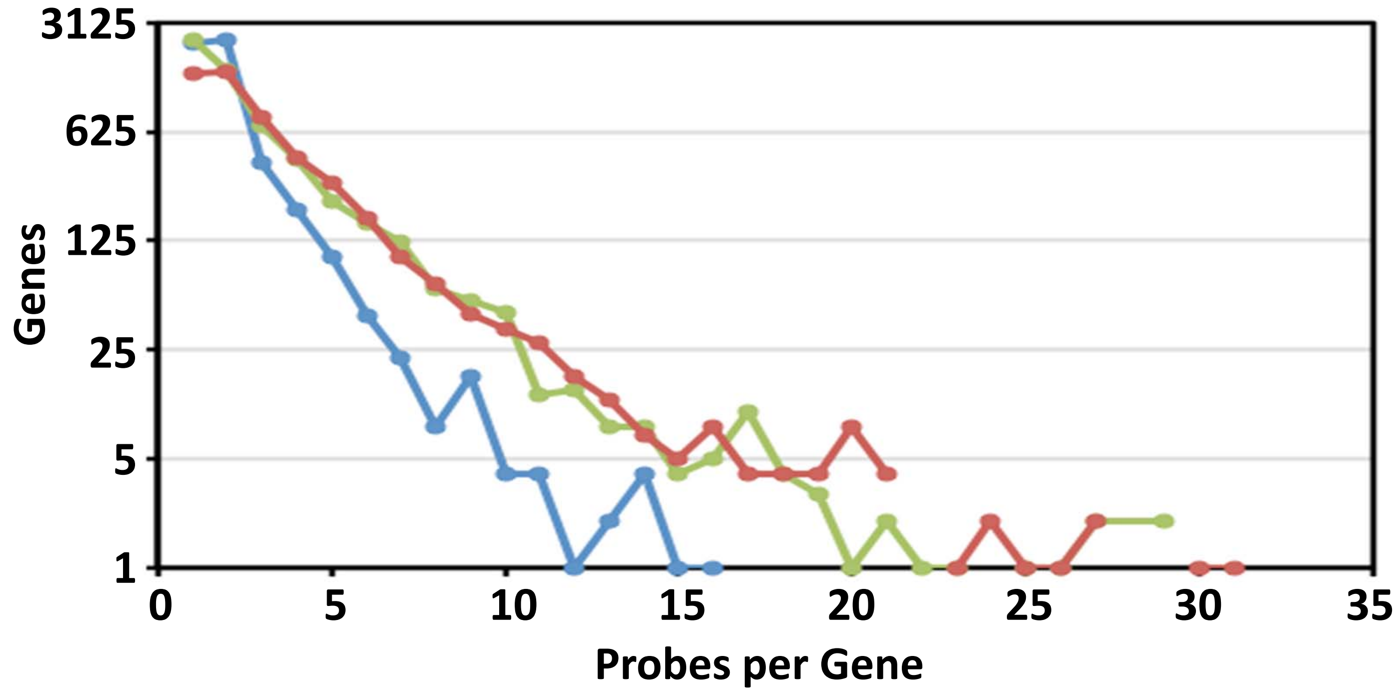

Additional File 3. Probes Per Gene. *P. falciparum* spotted 70mer array (blue), the *P. falciparum* Agilent 8x15K array (green), and the *P. berghei* Agilent 8x15K array (red).
