# Supplementary material for: New Agilent platform DNA microarrays for transcriptome analysis of Plasmodium falciparum and Plasmodium berghei for the malaria research community
Source: Malar J. 2012 Jun 8;11:187. doi: 10.1186/1475-2875-11-187 (PMC3411454; doi:10.1186/1475-2875-11-187)
Supplement: Additional file 4 — Figures and table describing array-wide technical reproducibility of hybridizations across three replicates. [file 1475-2875-11-187-S4.pdf]

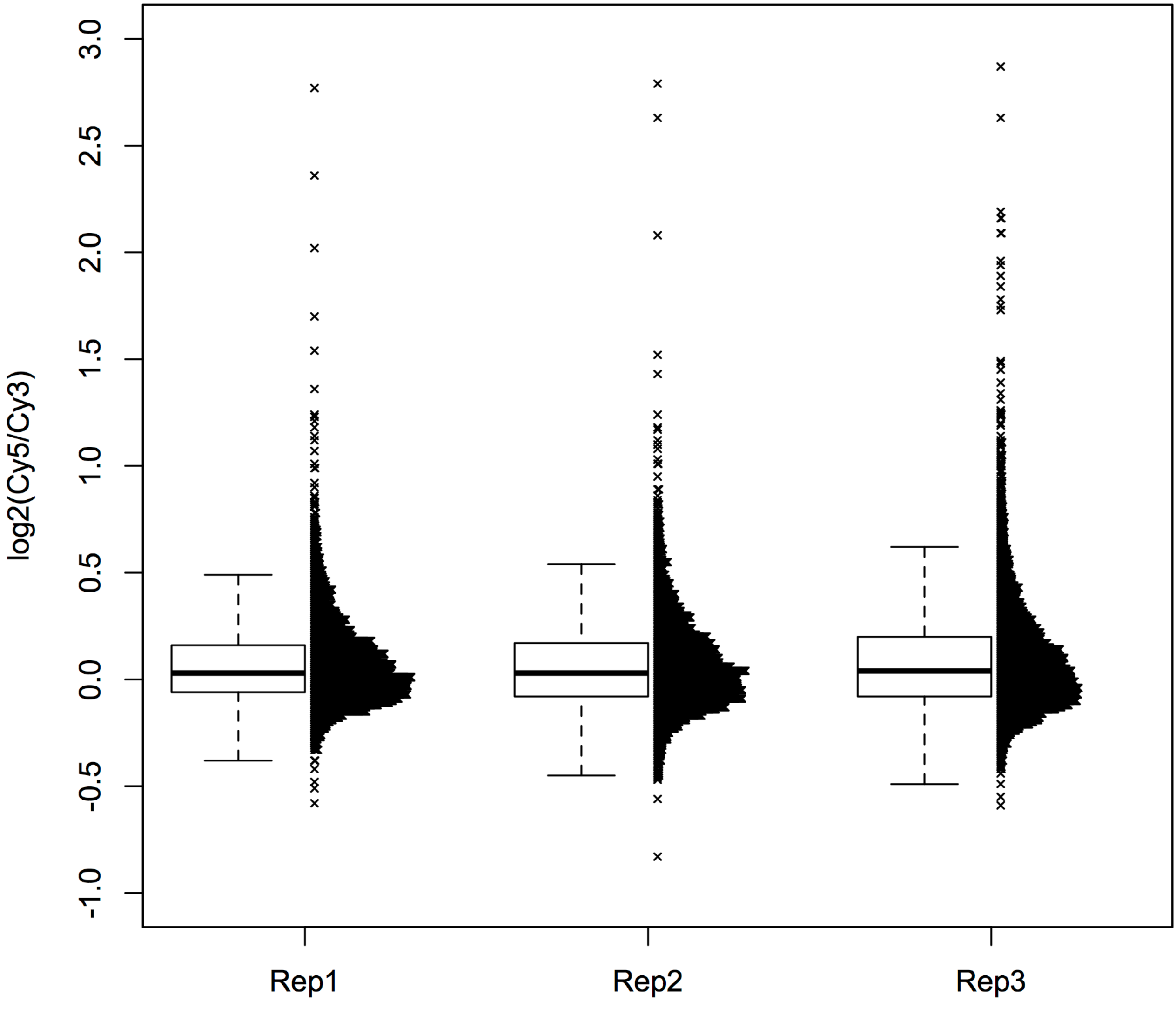


**Log2(Cy5/Cy3) Ratio by Gene**

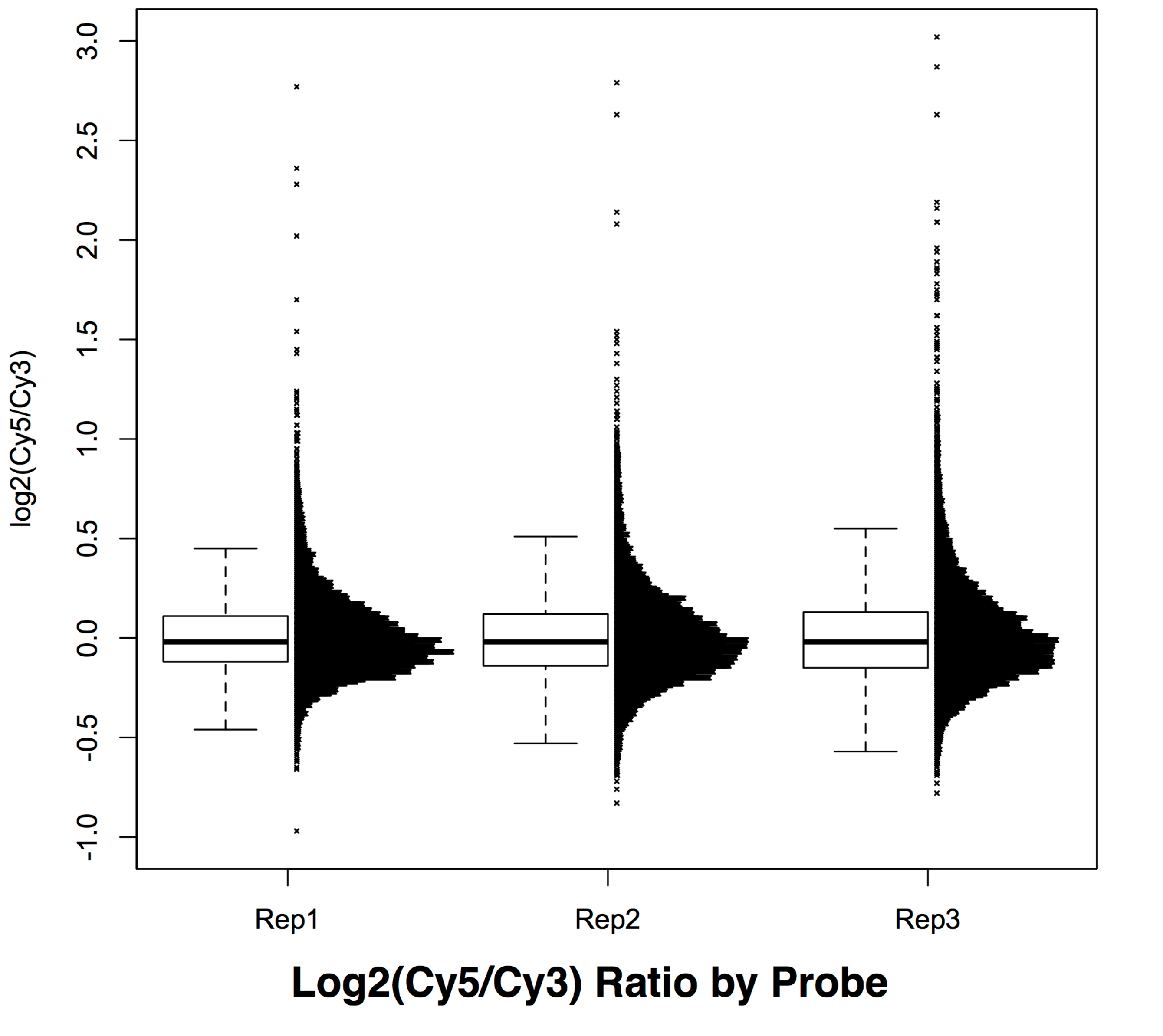

log2(Cy5/Cy3)
3.0
2.5
2.0
1.5
1.0
0.5
0.0
-0.5
-1.0
Rep1
Rep2
Rep3
Log2(Cy5/Cy3) Ratio by Probe

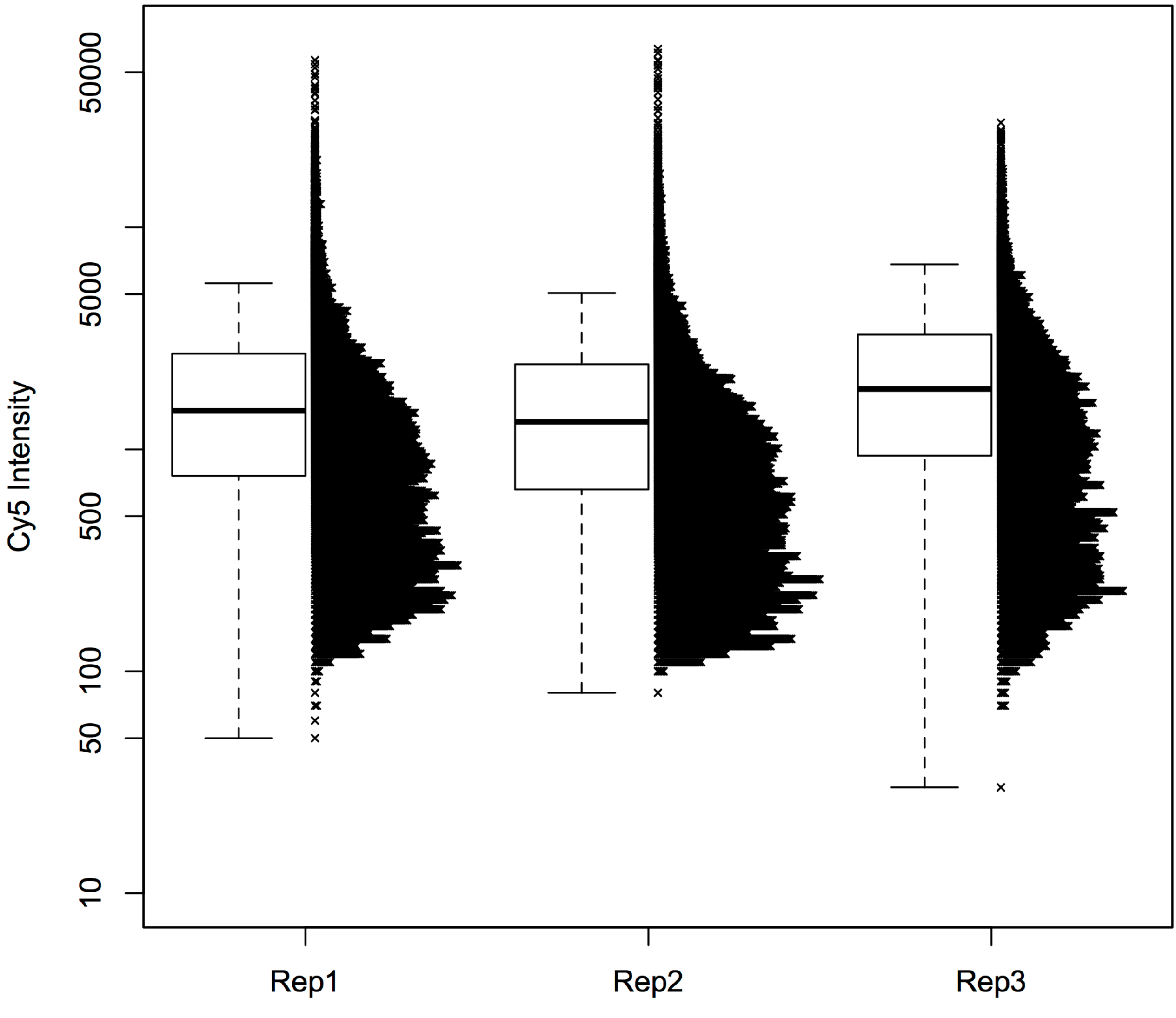


**Cy5 Intensity by Probe**

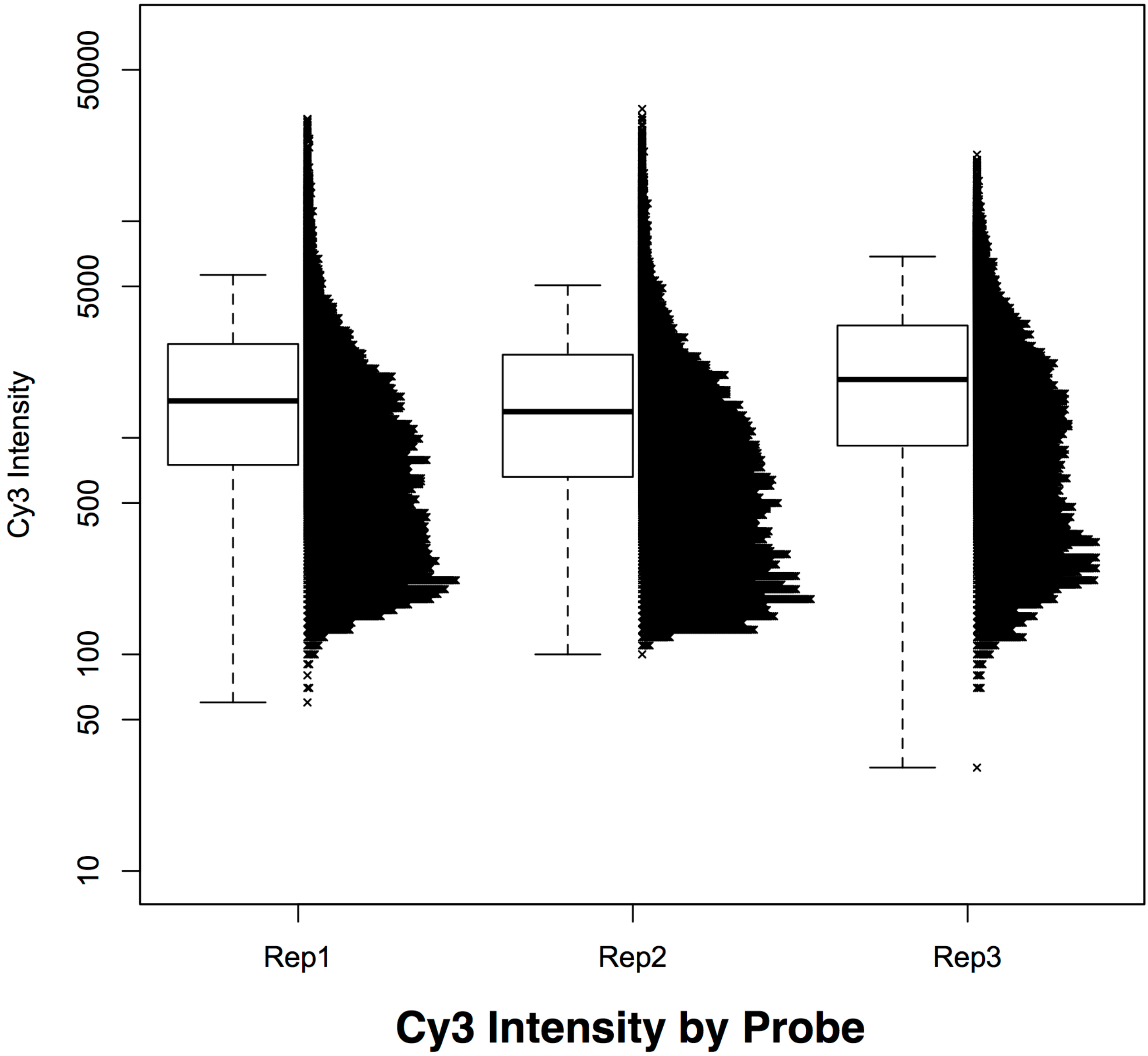
50000
5000
500
100
50
10
Cy3 Intensity
Rep1
Rep2
Rep3
Cy3 Intensity by Probe

| | Pearson's Coefficient of Correlation | | | |
|---|---|---|---|---|
| **Comparison** | **Gene log2(Cy5/Cy3)** | **Probe log2(Cy5/Cy3)** | **Probe Cy5 Intensity** | **Probe Cy3 Intensity** |
| Rep1 vs. Rep2 | 0.88 | 0.82 | 0.99 | 0.99 |
| Rep1 vs. Rep3 | 0.87 | 0.85 | 0.96 | 0.93 |
| Rep2 vs. Rep3 | 0.82 | 0.80 | 0.93 | 0.90 |

**Consistency of Technical Replicates.** Pearson's coefficient of correlation for comparisons of three replicates for at the gene- ($log_2$ ratio) and individual probe-level ($log_2$ ratio, Cy3 Intensity, Cy5 intensity).
