# Supplementary material for: New Agilent platform DNA microarrays for transcriptome analysis of Plasmodium falciparum and Plasmodium berghei for the malaria research community
Source: Malar J. 2012 Jun 8;11:187. doi: 10.1186/1475-2875-11-187 (PMC3411454; doi:10.1186/1475-2875-11-187)
Supplement: Additional file 5 — Table of titration series of total RNA starting amounts, resultant cDNA generation, dye-coupling, and array hybridization. [file 1475-2875-11-187-S5.pdf]

Input RNA (ng)	aminoallyl-cDNA (ng)	Dye-coupled cDNA (ng)	Dye Incorp. (fmol/ng)
500	463 +/- 13	241 +/- 4	17.2 +/- 0.4
2,000	630 +/- 26	393 +/- 3	14.9 +/- 0.5
5,000	928 +/- 12	655 +/- 4	30.1 +/- 0.4
10,000	1502 +/- 35	669 +/- 3	29.4 +/- 0.2

Efficacy of cDNA generation & dye-coupling. Table of titration series of total RNA starting amounts, resultant cDNA generation, dye-coupling, and array hybridization.
