# Supplementary material for: New Agilent platform DNA microarrays for transcriptome analysis of Plasmodium falciparum and Plasmodium berghei for the malaria research community
Source: Malar J. 2012 Jun 8;11:187. doi: 10.1186/1475-2875-11-187 (PMC3411454; doi:10.1186/1475-2875-11-187)
Supplement: Additional file 6 — Table of transcripts well-above background by amount hybridized and table of signal intensity correlation across a self-hybridization dilution series. [file 1475-2875-11-187-S6.pdf]

A

Amount hybridized	1000ng	500ng	250ng	100ng	50ng
**Transcripts well above background**	5254	5288	5219	5271	5199
**Percentage of unique transcripts represented**	91.3%	91.9%	90.7%	91.6%	90.4%

Average number of transcripts (n=3) with gene signal intensities called "Well above Backround".

B

Pearson's r of mean Cy3 intensity

Pearson's r of mean Cy5 intensity

	1000ng	500ng	250ng	100ng	50ng
**1000ng**		0.977	0.974	0.964	0.825
**500ng**	0.964		0.987	0.977	0.846
**250ng**	0.976	0.974		0.997	0.903
**100ng**	0.973	0.961	0.998		0.930
**50ng**	0.873	0.822	0.916	0.936	

Correlation of signal intensity across a self-hybridization dilution series . Pearson's coefficients of correlation between diminishing amounts of self-hybridized cDNA pools for Cy5 (bottom left) and Cy3 (top right) mean signal intensity (n=3) for 4810 genes.
